# Supplementary material for: Interleukin-4 Promotes Tuft Cell Differentiation and Acetylcholine Production in Intestinal Organoids of Non-Human Primate
Source: Int J Mol Sci. 2021 Jul 24;22(15):7921. doi: 10.3390/ijms22157921 (PMC8348364; doi:10.3390/ijms22157921)
Supplement: Supplementary file 1 [file ijms-22-07921-s001.zip › Final Revised1 Supplementary_Materials.pdf]

Supplementary Materials

Supplementary Figure S1. Proliferation and differentiation balances could be altered by culture media composition. a. Representative images of the organoids cultured under the original conditions using a p38 inhibitor (p38i) and under new conditions using IGF-1 and FGF-2 (Primary: day 2 primary culture, Passaged: day 6 passaged organoids). Both culture conditions generated organoids, however, changes in the number and morphology of organoids were observed when passaged. Scale bar, 500 μm. **b.** The number of organoids in each culture condition with the size over 150 μm were counted and the ratio is shown by the percentage (%). *p=0.003, Welch's t-test (n=4). **c.** Representative images of whole-mount immunohistochemistry of organoids cultured with indicated medium conditions (IF, DI, or IL-4; see details in Materials and Methods). Organoids were stained with antibodies against DCLK1 (green) and 5HT (red). It should be noted that these two markers were exclusive. DAPI was used to stain nuclei (blue). Arrowheads indicate DCLK1, and arrows indicate 5HT immunoreactive cells. Scale bar, 100 μm. **c.** Quantification of DCLK1-positive cells (DCLK1+) and 5HT-positive cells (5HT+) under the different culture conditions as shown in (b). The number of positive cells per 1.0×10^3 organoid cells is shown. Data were analyzed by ANOVA and Tukey's HSD tests (ANOVA $p < 0.001$ (DCLK1) and p=0.02 (5HT)) in the DCLK1+ (a or b, $p < 0.001$) and 5HT+ (a′ or b′, p=0.018), respectively (n=3).

Supplementary Figure S2. Statistical analysis of genes preferentially expressed in IL-4-induced macaque organoids. Volcano plot analysis of the -log10 (FDR) plotted against log2 (fold changes) is shown. Red and blue plots represent the genes preferentially expressed in the culture medium supplemented with or without IL-4, respectively. (FC ≥ |1.5|, FDR ≤ 0.01; binominal (Wald) test with Benjamin-Hochberg correction).

Supplementary Figure S3. Transcriptional analysis of macaque intestinal organoids cultured with IL-13. a. Immunostaining of organoids with anti-DCLK1 (green) and anti-5HT (red) antibodies with (lower column) or without (upper column, DI) IL-13 treatment for 72 h. Arrowheads indicate DCLK1 and arrows indicate 5-HT immunoreactive cells. Scale bar, 100 μm. **b-c.** RNA-seq was performed as described in the Materials and Methods section. (**b**) Principal component analysis (PCA) for the transcriptome profiles of the organoids cultured with DI and IL13 conditions. (**c**) A heatmap shows 30 tuft cell marker genes which highly expressed in the organoid induced by IL-13. Color bars are relative expression levels based on row-wise z-score of transformed TPM values.

Supplementary Figure S4. IL-4 supplementation induces goblet and Paneth cells in addition to tuft cells. Micrographs of whole-mount immunohistochemistry of intestinal organoids cultured in three different medium compositions: maintenance media (IF), differentiation media (DI), or differentiation media with Interleukin-4 (IL-4). Organoids were stained with (a) anti-MUC2 antibodies (green) and (b) with anti-DEFA6 antibodies (green). DAPI was used to stain the nuclei (blue). Arrowheads indicate immunoreactive cells. Scale bar, 100 μm.

Supplementary Figure S5. ChAT expression analysis by immunohistochemistry using macaque intestinal tissue. The immunohistochemical analysis of native tissue using ant-5HT (green) and anti-CHAT (red) antibodies indicated that 5-HT and **ChAT** expression was exclusive. The nuclei were stained with DAPI. Arrowheads indicate 5-HT and arrows indicate **ChAT** immunoreactive cells. The lower panels show enlarged views of squares in upper panels. Scale bar, 50 μm.

Video S1. The effect of Ach on mouse intestinal organoids. Ach stimulation induce granule release from apical side of the organoid.
