# Supplementary material for: Interleukin-4 Promotes Tuft Cell Differentiation and Acetylcholine Production in Intestinal Organoids of Non-Human Primate
Source: Int J Mol Sci. 2021 Jul 24;22(15):7921. doi: 10.3390/ijms22157921 (PMC8348364; doi:10.3390/ijms22157921)

Supplementary Figure S1

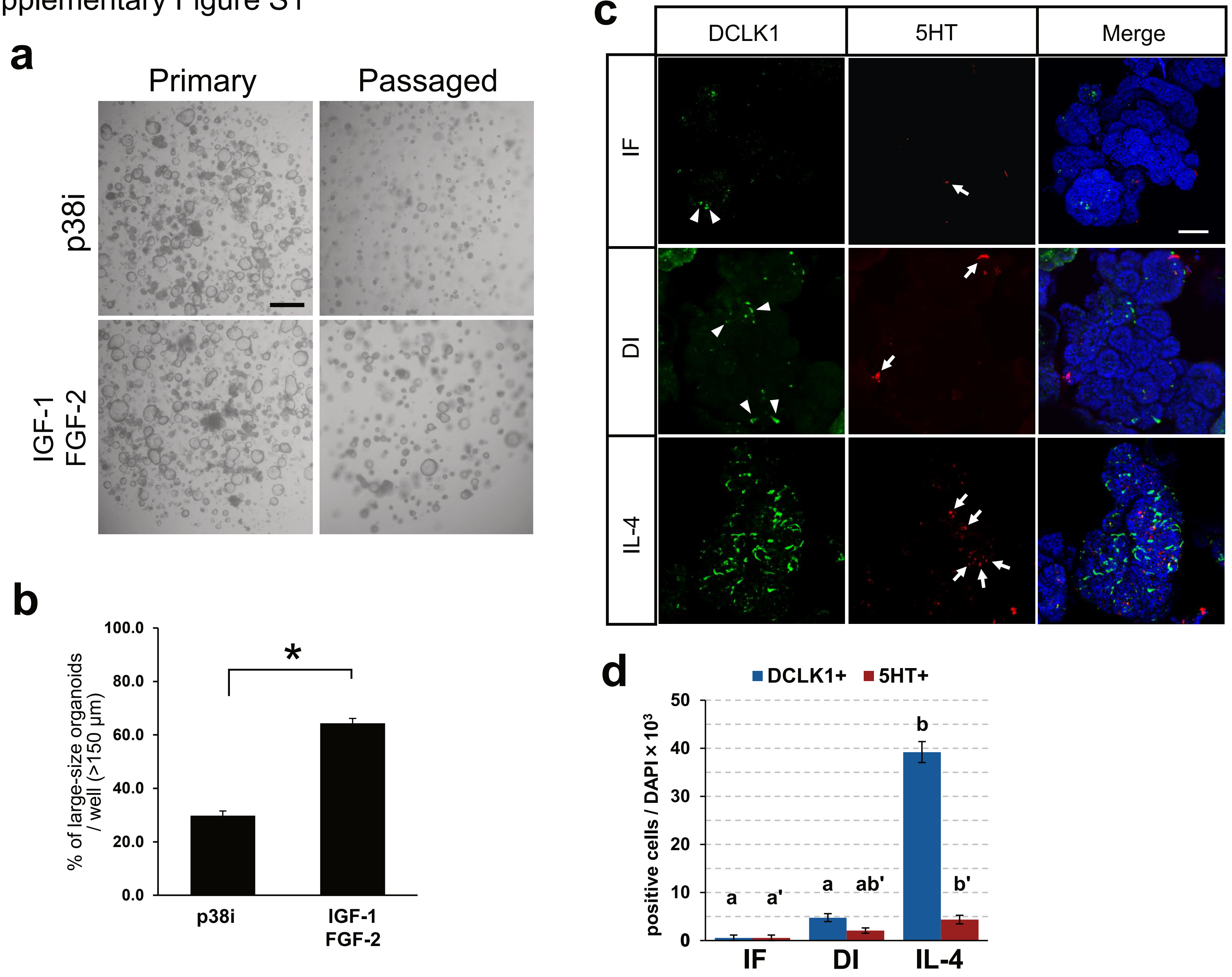

Supplementary Figure S2

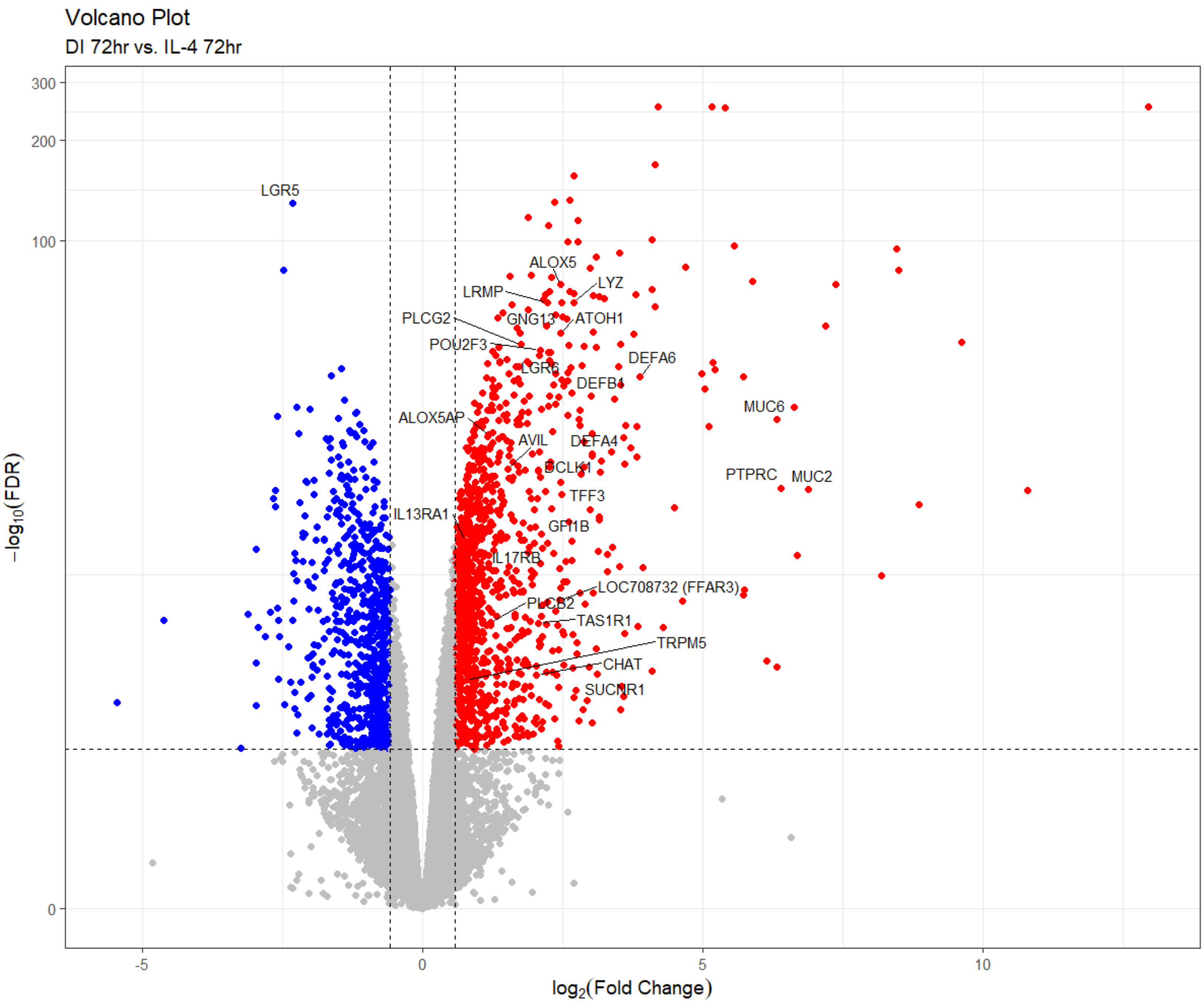

Supplementary Figure S3

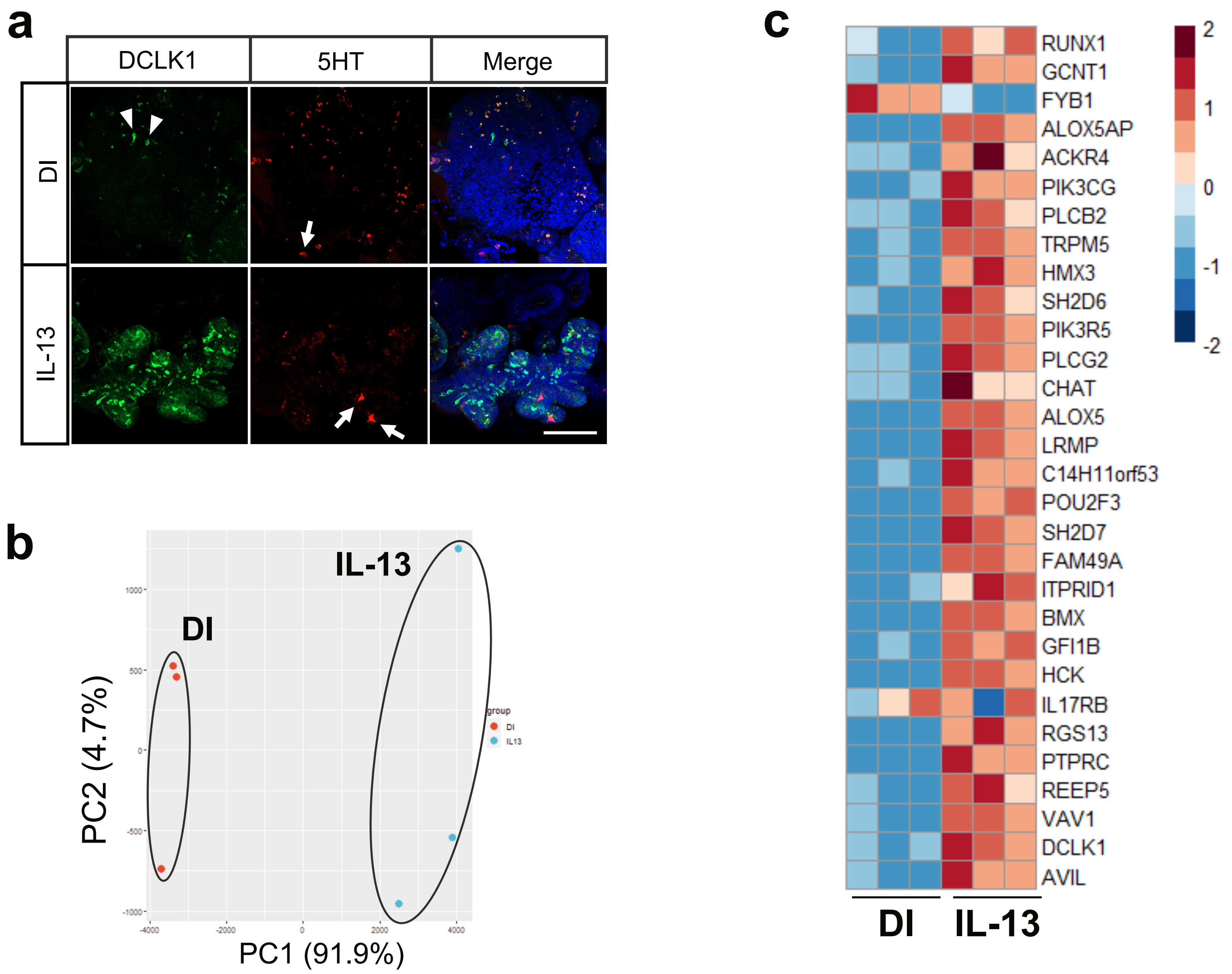

Supplementary Figure S4

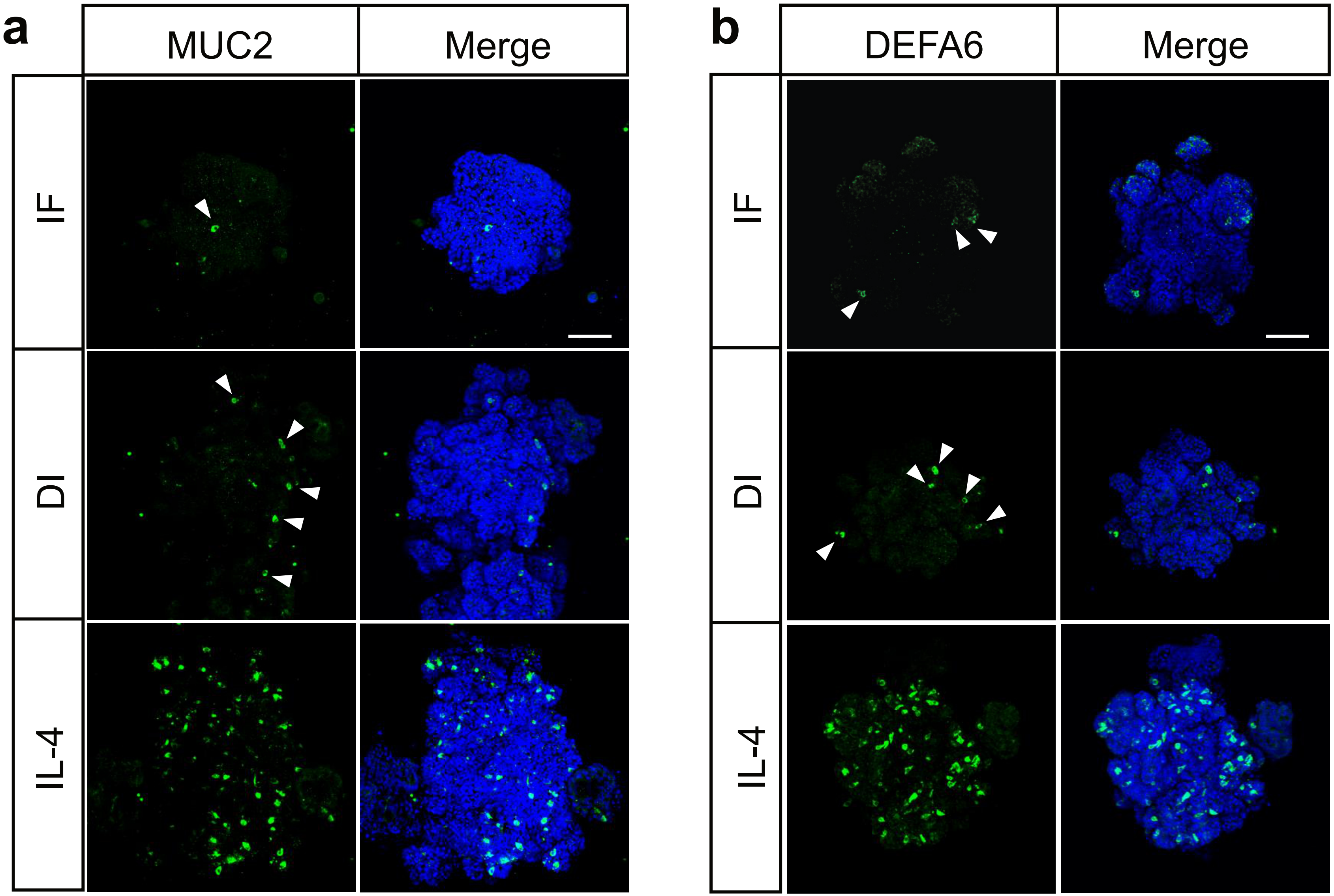

Supplementary Figure S5

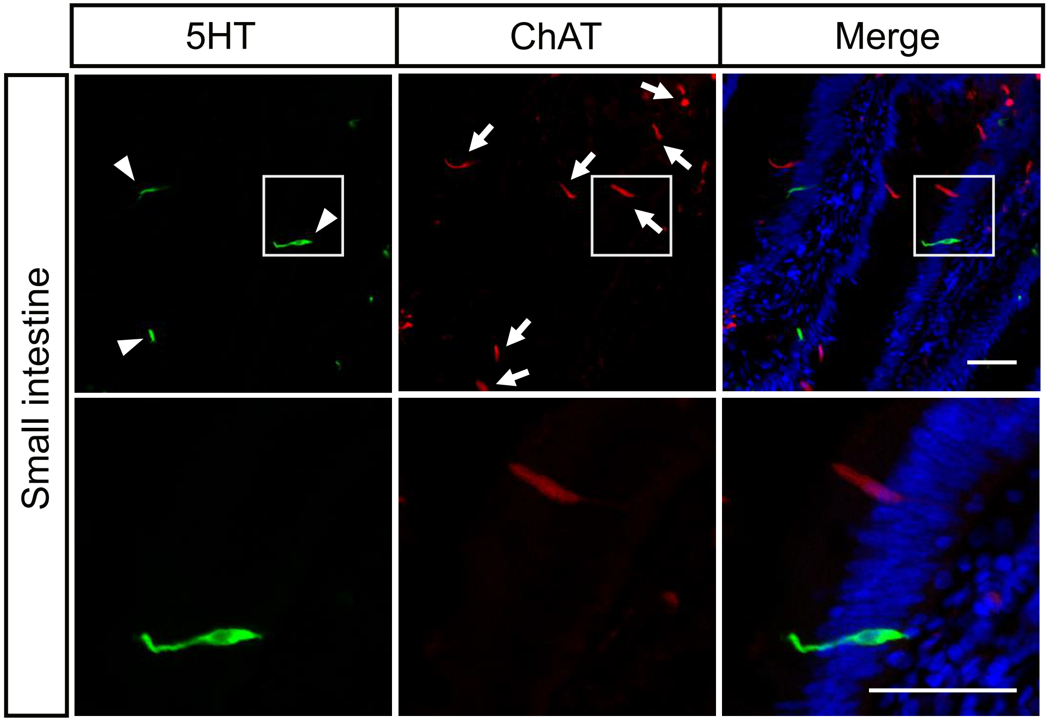

Supplement: Supplementary file 1 [file ijms-22-07921-s001.zip › Revised Supplementary Figure.pdf]
